# TRANSLATION, CULTURAL ADAPTATION AND VALIDATION OF THE FOOT FUNCTION INDEX - REVISED (FFI-R)

**DOI:** 10.1590/1413-785220172505172107

**Published:** 2017

**Authors:** KELLY CRISTINA STÉFANI, MIGUEL VIANA PEREIRA, PEDRO RIZZI OLIVEIRA, Paloma Yan Lam Wun

**Affiliations:** 1. Hospital do Servidor Público Estadual de São Paulo, Departamento de Ortopedia e Traumatologia, Grupo de Pé e Tornozelo, São Paulo, SP, Brazil.

**Keywords:** Surveys and questionnaires, Translating, Foot diseases, Ankle injuries

## Abstract

**Objective:**

The aim of this study was to translate, culturally adapt, and validate the “Foot Function Index - Revised” (FFI-R) for use in Brazilian Portuguese.

**Methods:**

The scale was translated and administered (as recommended by Guillemin, 2000) to 52 patients in the postoperative period after foot and ankle surgery. Seven days after the initial assessment, the scale was readministered by a different interviewer. The data were entered into an Excel spreadsheet and analyzed using SPSS version 23.0 software for Mac. Reproducibility was assessed using intraclass correlation analysis.

**Results:**

were considered statistically significant at a type I error rate of 5%. Results: The following random-effects intraclass correlation coefficients (ICC) were obtained for each score on the FFI-R: 0.625 for pain, 0.558 for stiffness, 0.757 for difficulty, 0.718 for activity restrictions, 0.854 for personal concerns, and 0.753 for the total score.

**Conclusion:**

The FFI-R was successfully translated to Portuguese and culturally adapted for use in Brazilian patients, demonstrating satisfactory validity and reliability. **Level of Evidence I, Testing of Previously Developed Diagnostic Criteria on Consecutive Patients (with universally applied reference “golg” standard).**

## RESUMO

**Objetivo:**

O objetivo deste estudo traduzir, fazer a adaptação cultural e a validação do “Foot Function Index - Revised” (FFI-R) para o idioma português.

**Métodos:**

A escala foi traduzida e aplicada (segundo recomendado por Guillemin, 2000) a 52 pacientes depois de cirurgia do pé e tornozelo. Sete dias depois da avaliação inicial, a escala foi novamente aplicada por outro entrevistador. Os dados foram inseridos em uma planilha do Excel e a análise estatística foi realizada no software SPSS 23.0 para Mac. A análise de correlação intraclasse foi realizada para avaliar a reprodutibilidade. Os resultados foram considerados estatisticamente significantes em erro do tipo I de até 5%.

**Resultados:**

Foram obtidos os seguintes coeficientes de correlação intraclasse (CCI) de efeitos aleatórios para cada pontuação no FFI-R: 0,625 para dor, 0,558 para rigidez, 0,757 para dificuldade, 0,718 para restrição de atividades, 0,854 para preocupações pessoais e 0,753 para o escore total.

**Conclusão:**

O FFI-R foi traduzido com sucesso para o português e adaptado culturalmente para aplicação em pacientes brasileiros, demonstrando validade e confiabilidade satisfatórias. **Nível de Evidência I, Teste de Critérios Diagnósticos Desenvolvidos Anteriormente em Pacientes Consecutivos (com padrão de referência "ouro" aplicado).**

## INTRODUCTION

The use of assessment scales in scientific studies is an essential requirement for the comparison of different treatments in patients with the same diagnosis.^1^ ^-^ ^9^ The majority of outcome assessment scales are developed in English and directed at patients who speak this particular language. As a result, they must be translated and culturally adapted in order to be used in any other country. The statistical properties of the adapted instrument must then be evaluated based on published criteria to ensure its equivalence to the original instrument.^10^ ^,^ ^11^ The aim of this study was to translate, adapt and validate the “Foot Function Index - Revised” (FFI-R) for use in Brazilian Portuguese.^12^

The FFI was developed to measure the impact of the pain, disability and activity restriction associated with foot pathology on patient functioning. It is a self-administered instrument composed of 23 items divided into three subscales.^1^ ^,^ ^13^ ^-^ ^15^ The FFI has already been translated, culturally adapted and validated for use in Brazilian Portuguese.^16^

The FFI-R was developed at a later date in response to criticism of the original scale. After the unidimensionality of the FFI-R was confirmed by an analysis of its subscales, responses were coded into four categories for ease of use. The FFI is a pioneer instrument in the patient-centered measurement of foot health, and is widely used throughout the world. Its use of concrete indicators to provide a reliable measure of foot health introduced an important paradigm shift from subjective to objective measurements in the area of clinical foot assessments. The coding of the FFI-R into four response categories facilitated its use in the assessment of foot health.^3^ ^,^ ^12^

## MATERIALS AND METHODS 

The translation and cultural adaptation processes were carried out in five stages, as recommended by the literature:^10^ ^,^ ^11^ ^,^ ^17^ a) stage 1 (translation): the FFI-R was first translated to Portuguese by two independent Brazilian translators, one of whom was an official translator, while the other was a technical translator with expertise in health care. Both translators were aware of the purpose of the study; b) stage 2 (synthesis): the translations were compared and discussed with the translators. When disagreements arose, changes were made as required until a consensus was reached (Portuguese version 1); c) stage 3 (back translation to English): the first Portuguese version of the scale was translated to English by two native American translators blind to the purpose of the study; d) stage 4 (expert committee review): a meeting was scheduled with all four translators to produce a “pre-final” version of the scale; e) stage 5: cultural adaptation: the pre-final version of the questionnaire was administered to 52 patients aged 18 years and older. The version was considered final when all items were judged as “not understood” by less than 10% of the sample.^1^ ^-^ ^4^ ^,^ ^12^

The inclusion criteria were late postoperative period (at least 12 months) after foot or ankle surgery at the Foot and Ankle Department of the Hospital do Servidor Público Estadual (HSPE), and absence of medication use or additional procedures for one week after the administration of the pre-final version of the questionnaire to ensure reproducibility. The presence of cognitive impairments which could interfere with the administration of the questionnaire was the only exclusion criterion.

The sociocultural characteristics of the 52 patients in the late postoperative period after foot and ankle surgery who participated in the reproducibility and validation studies of the Portuguese version of the FFI-R were as follows: 39 were female (75%), and 13 were male (25%); mean age was 56 years (range: 39 to 81 years); mean length of postoperative period was 4 years (range: 1 to 11 years); 22 had completed secondary education, 29 had a university degree, and one had gone to graduate school. Study approved in the Brazilian Platform CAAE: 49066915.9.0000.5463 under Opinion constituted 1,283,807.

### Reproducibility and validity of the portuguese version of the FFI-R

The reproducibility of the Portuguese version of the FFI-R was evaluated in a sample of 52 patients in the late postoperative period after foot or ankle surgery. The scale was administered by a previously trained interviewer (interviewer 1). After a seven day period, a new assessment was conducted by interviewer 2.

Data were entered into an Excel® spreadsheet and analyzed using SPSS version 23.0 for MAC. The mean and standard deviation of each item in the Brazilian version of the FFI-R were calculated. The relationship between the assessments was evaluated by linear correlation analysis followed by paired comparisons of scores on the first and second evaluations. This procedure was performed using non-parametric methods due to the skewed distribution of the data. Lastly, reproducibility was assessed using intraclass correlation (ICC) analysis. Results were considered statistically significant at a type I error rate of 5%.

## RESULTS

When the pre-final version of the questionnaire was administered to the validation sample in the cultural adaptation stage, no item reached the 10% comprehension threshold, and as such, the instrument was deemed culturally appropriate. The final version of the FFI-R in Portuguese is presented in the Appendix 1. The mean time of questionnaire administration was 20 minutes, and the interval between the two assessments was seven days.

The mean ± SD of pain scores on the first and second assessment were 44.46%+21.36 and 39.21%+18.36, respectively. The Spearman correlation between these values was 0.674, significant at *p*<0.001. The two scores did not significantly differ according to Wilcoxon’s paired t-test, *p*=0.06. The random-effects intraclass correlation coefficient (ICC) corresponding to the test-retest reliability of this particular score was 0.625 [95%CI 0.428 to 0.766], *p*<0.001. (Table 1)

**Table 1:** Random-effects intraclass correlation coefficient (ICC) for pain scores.

	Intraclass correlation	95% Confidence interval			F Test with true value 0		
		Lower bound	Upper bound	Value	df1	df2	Sig
Single measures	.625	.428	.766	4,338	51	52	.000

The mean ± SD of stiffness scores on the first and second assessment were 39.00%±20.54 and 38.96%±17.40, respectively. The Spearman correlation between these values was 0.513, significant at *p*<0.001. The two scores did not significantly differ according to Wilcoxon’s paired t-test, *p*=0.06. The random-effects ICC of the stiffness score was 0.558 [95%CI 0.340 to 0.719], *p*<0.001.

The mean ± SD of difficulty scores on the first and second assessment were 44.47%±28.33 and 39.81%±24.01, respectively. The Spearman correlation between these values was 0.754, significant at *p*<0.001. The two scores did not significantly differ according to Wilcoxon’s paired t-test, *p*=0.06. The random-effects ICC of the FFI-R difficulty score was 0.745 [95%CI 0.595 to 0.845], *p*<0.001.

The mean ± SD of activity limitation scores on the first and second assessment were 41.35%±23.29 and 40.97%±21.05, respectively. The Spearman correlation between these values was 0.756, significant at *p*<0.001. The two scores did not significantly differ according to Wilcoxon’s paired t-test, *p*=0.06. The random-effects ICC of the activity limitation score was 0.718 [95%CI 0.556 to 0.827], *p*<0.001.

The mean + SD of social activity scores on the first and second assessment were 36.44%±23.64 and 39.95%±19.57, respectively. The Spearman correlation between these values was 0.691, significant at *p*<0.001. The two scores did not significantly differ according to Wilcoxon’s paired t-test, *p*=0.06. The random-effects ICC of the social functioning score was 0.854 [95%CI 0.700 to 0.913], *p*<0.001.

The mean ± SD of total scores on the first and second assessment were 41.01%±4.23 and 39.01%±0.09, respectively. The Spearman correlation between these values was 0.760, significant at *p*<0.001. The two scores did not significantly differ according to Wilcoxon’s paired t-test, *p*=0.06. The random-effects ICC of total scores on the FFI-R was 0.793 [95%CI 0.667 to 0.876], *p*<0.001.

## DISCUSSION

When assessing the outcome of orthopedic treatments, there is often significant concern about the impact of the intervention on the patient’s quality of life, emotional well-being, and performance in daily activities.^18^

Two main challenges are often faced in the assessment process: one concerns the quantification of subjective information and the selection of questions for assessment instruments, while the other involves the administration of these questionnaires in different countries to allow for cross-cultural comparisons.^19^ ^,^ ^20^ These instruments are usually developed in English, and must therefore be translated and analyzed for their statistical properties prior to being used in any other cultural context.^1^ ^,^ ^2^ ^,^ ^10^ ^,^ ^11^

In the present study, no comprehension issues were encountered, since all items in the questionnaire refer to patients’ daily activities. In the cultural adaptation stage, no item reached the 10% comprehension threshold, and as such, the pre-final version of the FFI-R was deemed culturally appropriate.

The reproducibility of the Portuguese version of the FFI-R was evaluated in a sample of 52 patients in the late postoperative period after foot or ankle surgery. The scale was first administered by a previously trained researcher (assessment 1), then readministered by another interviewer (assessment 2). Scores on assessments 1 and 2 did not significantly differ from one another and were significantly correlated, which speaks to the reliability of the instrument. The Portuguese version of the FFI-R was also shown to have strong internal consistency, as evidenced by intraclass correlation analysis. (Tables 1 to 6)

**Table 2:** Random-effects intraclass correlation coefficient (ICC) for stiffness scores.

	Intraclass correlation	95% Confidence interval		F Test with true value 0			
		Lower bound	Upper bound	Value	df1	df2	Sig
Single measures	.558	.340	.719	3,522	51	52	.000

**Table 3:** Random-effects intraclass correlation coefficient (ICC) for difficulty scores.

	Intraclass correlation	95% Confidence interval		F Test with true value 0			
		Lower bound	Upper bound	Value	df1	df2	Sig
Single measures	.745	.595	.845	6,833	51	52	.000

**Table 4:** Random-effects intraclass correlation coefficient (ICC) for activity limitation scores.

	Intraclass correlation	95% Confidence interval		F Test with true value 0			
		Lower bound	Upper bound	Value	df1	df2	Sig
Single measures	.718	.556	.827	6,083	51	52	.000

**Table 5:** Random-effects intraclass correlation coefficient (ICC) for social functioning scores.

	Intraclass correlation	95% Confidence interval		F Test with true value 0			
		Lower bound	Upper bound	Value	df1	df2	Sig
Single measures	.854	.760	.913	12,715	51	52	.000

**Table 6:** Random-effects intraclass correlation coefficient (ICC) for total scores on the FFI-R.

	Intraclass correlation	95% Confidence interval		F Test with true value 0			
		Lower bound	Upper bound	Value	df1	df2	Sig
Single measures	.793	.667	.876	8,678	51	52	.000

## CONCLUSION

The FFI-R was successfully translated and culturally adapted for use in Brazilian patients, demonstrating satisfactory validity and reliability.
